# Hay-Fever

**Published:** 1870-07

**Authors:** 


### Hay-Fever.

ITS SYMPTOMS, CAUSES, AND MEANS OF RELIEF.

From the first of July to the ending of October we have an epidemic in this valley, known among the people as Hay-Fever. The first intimation the patient has of its presence, is a constant disposition to sneeze, with a tickling sensation about the fauces and posterior naries. In a day or two after the appearance of these symptoms, the eye-lids become swollen—the eyes appear red and inflamed, discharging hot water, which irritates the cheek wherever it touches. There is a dull, heavy feeling about the forehead—hot skin—moderate pulse—aching pains in the back and limbs, and a feeling of great lassitude, with impairment of strength. Occasionally a disagreeable, hacking cough accompanies these symptoms, with a sensation of some foreign substance in the trachea, or windpipe.

Hay-fever is supposed to be caused by an introduction of minute hay and other seeds, upon the mucous surfaces of the throat, nose and eyes. These little seeds are floating about in the atmosphere at this season of the year, entering the breathing apparatus, eyes and nose of every one, and producing their irritating effect upon those most susceptible to such slight annoyances. Usually, those who suffer from catarrh in any form, are the subjects of hay-fever, as the mucous membrane of throat and nose are already in a state of irritation, which is readily increased by the presence of any foreign substance.

The treatment is as simple as it is efficacious. Usually a week's sojourn by the sea-side will dispel all the disagreeable symptoms—as the patient is then removed from the exciting cause, and receives the soothing influence of the sea breeze. A friend, whose wife is yearly afflicted with hay-fever, informs me that the sneezing and painful sensation about the eyes and forehead stops the moment she enters the ferry-boat at Jersey City; and that all the symptoms leave her in less than a week after her arrival at the sea-side. We know of two or three other instances, where the relief was quite as instantaneous upon their arrival in the air from salt water.

To those who cannot afford to engage in this somewhat expensive, but exceedingly pleasant "treatment," we recommend the following means of medication:

Procure an ounce of the *wine of opium;* place it in one pint of boiling water—saturate cloths with this mixture, and apply it over the nose, eyes, and forehead, as hot as can be borne. If the symptoms are severe, continue the applica tion during the entire day, and at night add to the preparation of opium a teaspoonful of common table-salt, place this in an old tea-kettle, under which have a brisk fire—then inhale the medicated steam through the kettle-spout. Repeat the fomentations and inhalations the following day, if necessary.

Small particles of ice frequently taken into the mouth, will relieve the heat, dryness and tickling often felt in the roof of the mouth.—Glycerine, applied to the nostrils by means of a camel-hair brush, will give relief from sneezing at night.

If the bowels are constipated, a sidlitz powder should be taken, occasionally, in the morning, before eating.

Bathe the eyes frequently in cold, soft water, in which some table-salt has been dissolved—about a teaspoonful of salt to a pint of clear water. This will relieve the itching, smarting and watering of the eyes. Live on wholesome food—avoid tea and coffee—use chocolate instead. Pastry should not be indulged in. Roast beef and baked potatoes, and plenty of it, is the most wholesome diet.

If the disease continues beyond a week or two, a nasal douche should be procured, and from ten to thirty drops of the solution of carbolic acid should be thoroughly agitated in a pint of luke-warm water, and the solution passed through the nostrils by means of the Douche, every morning and evening. This will arrest the disease in its chronic or aggrivated form. Frequent bathing in salt water, with friction to the skin, by means of a crash towel, vigorously applied by continued rubbing, is a very important part of the treatment.

If our instructions are faithfully followed, we believe every person subject to the annoyance of "hay-fever" has within his reach the means of self-cure.

